# A Randomized Evaluation of a Demand Creation Lottery for Voluntary Medical Male Circumcision Among Adults in Tanzania

**DOI:** 10.1097/QAI.0000000000001042

**Published:** 2016-10-06

**Authors:** Eva Bazant, Hally Mahler, Michael Machaku, Ruth Lemwayi, Yusuph Kulindwa, Jackson Gisenge Lija, Baraka Mpora, Denice Ochola, Supriya Sarkar, Emma Williams, Marya Plotkin, James Juma

**Affiliations:** * Jhpiego, Baltimore, MD;; † FHI 360, Washington, DC (formerly Jhpiego, Dar es Salaam, Tanzania);; ‡ Jhpiego, Dar es Salaam, Tanzania;; § Ministry of Health, Community Development, Gender, the Elderly and Children, Dar es Salaam, Tanzania;; ‖ Jhpiego/Consultant, Baltimore, MD; and; ¶ Department of Epidemiology, Rollins School of Public Health, Laney Graduate School, Emory University, Atlanta, GA.

**Keywords:** voluntary medical male circumcision, Tanzania, HIV, AIDS, demand creation

## Abstract

Uptake of voluntary medical male circumcision (VMMC) among adult men has fallen short of targets in Tanzania. We evaluated a smartphone raffle intervention designed to increase VMMC uptake in three regions.

**Methods:**

Among 7 matched pairs of health facilities, 1 in each pair was randomly assigned to the intervention, consisting of a weekly smartphone raffle for clients returning for follow-up and monthly raffle for peer promoters and providers. VMMC records of clients aged 20 and older were analyzed over three months, with the number performed compared with the same months in the previous year. In multivariable models, the intervention's effect on number of VMMCs was adjusted for client factors and clustering. Focus groups with clients and peer promoters explored preferences for VMMC incentives.

**Results:**

VMMCs increased 47% and 8% in the intervention and control groups, respectively; however, the changes were not significantly different from one another. In the Iringa region subanalysis, VMMCs in the intervention group increased 336% (exponentiated coefficient of 3.36, 95% CI: 1.14 to 9.90; *P* = 0.028), after controlling for facility pair, percentage of clients ≥ age 30, and percentage testing HIV positive; the control group had a more modest 63% increase (exponentiated coefficient 1.63, 95% CI: 1.18 to 2.26; *P* = 0.003). The changes were not significantly different. Focus group respondents expressed mixed opinions about smartphone raffles; some favored smaller cash incentive or transportation reimbursement.

**Implications:**

A smartphone raffle might increase VMMC uptake in some settings by helping late adopters move from intention to action; however, this study did not find strong evidence to support its implementation broadly.

## BACKGROUND

Voluntary medical male circumcision (VMMC) reduces female-to-male HIV transmission by approximately 60%.^1–4^ In response to 2007 guidance from the World Health Organization and UNAIDS,^5^ 14 sub-Saharan African countries aim to scale up VMMC to 80% of males aged 15–49.^6^ By late 2014, nearly 9.1 million men had been circumcised.^7,8^ Rapid scale-up of VMMC could avert hundreds of thousands of HIV infections and save billions of dollars.^6^

In Tanzania, the adult HIV prevalence rate was 5.1% in 2011–2012.^9^ In 12 of Tanzania's 34 regions, free VMMC services are available to males aged 10–34. Preadolescence is the preferred time for circumcision; among adults, the popularity of seeking VMMC is low. From 2009 to 2014, only 21% of VMMC clients were men aged 25–34 in Iringa, Njombe, and Tabora regions (16,252/76,813). In Iringa, men and women report that seeking VMMC is shameful for adult and married men.^10^ A demand-creation strategy developed in 2012 to increase adult VMMC uptake included revising behavior change materials and messages, establishing a peer promoter program, advocating with business and religious leaders in communities, and modifying services (eg, to allow adults to be served first). Between 2012 and 2013, these efforts led to only a 5% increase in VMMCs among men ages 20 and older. The government has identified a need to increase VMMC uptake by males aged 20–34.^11^

Financial incentives have been investigated across a variety of preventive health services including those related to sexually transmitted infections (STIs).^12–14^ Incentives were shown to increase VMMC service uptake in Kenya and testing for HIV and other STIs.^15–18^

The use of lotteries is a promising strategy to increase the motivation of beneficiaries to use health services.^19^ Lotteries are believed to enhance targeting by introducing an element of risk, which appeals to risk-taking individuals, such as those at greater risk of acquiring HIV. Studies have found a tendency to overestimate the chance of winning a big prize even when the likelihood of winning is small, and a preference for a small chance at a large reward over the certainty of a small reward.^20–22^ To our knowledge, no studies have addressed the use of lotteries to increase the delivery of VMMC services.

The Government of Tanzania's VMMC services have been supported by Jhpiego since 2009 with funding from USAID through The President's Emergency Plan For AIDS Relief (PEPFAR). Routine services at fixed facilities are supplemented by frequent outreach activities. From 2009 to 2012, the male circumcision prevalence rate among males aged 10–34 increased from 29% to 60% in Iringa, from 29% to 49% in Njombe, and from 38% to 56% in Tabora.^23,24^ In 2011–12, HIV prevalence was 6.9%, 14.2%, and 4.5% in Iringa, Njombe, and Tabora regions, respectively.^9^ In this study, lottery messages portrayed the adult man who received VMMC as a socially successful *Bwana Mkubwa* (“big man” in Swahili) to try to reduce stigma associated with seeking circumcision services in adulthood. In Tanzania, 61% of the population owns a mobile telephone.^9^ The smartphone was selected as the incentive based on the idea that mobile phone ownership conveys social status, which is supported by research from Kenya.^25^ The intervention, agreed upon with local stakeholders, drew on the Health Belief Model,^26^ in which barriers to action including stigma are believed to be overcome through motivational incentives.

This article describes the evaluation of the smartphone lottery and its effects on the volume of VMMC clients aged 20 years and above. We hypothesized that the smartphone raffle incentive would increase by net 20% the number of VMMC clients aged 20 and above at routine service delivery, and be an acceptable incentive among clients and peer promoters at the intervention and control sites.

## METHODS

### Study Design and Sample

**FIGURE 1.**
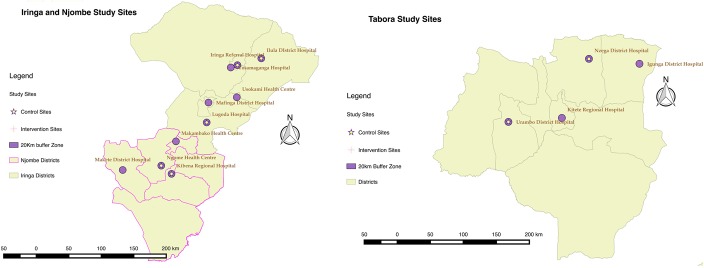
Study sites by study group and region.

The study design was a randomized evaluation using mixed (quantitative and qualitative) methods. From November 2014 to February 2015, 7 pairs of facilities were selected from 17 available facilities in the three regions. Facilities were matched on region, patient volume, and facility type. There were 2 exceptions. A hospital was matched to a large health center in Iringa. Also, a health center in Iringa was matched to a health center in in Njombe. One facility in each pair was randomly allocated in a coin toss to intervention or control, with the selection made by government stakeholders (Fig. 1).

### Program Elements

At all facilities, mass media campaigns and peers promoted VMMC services (Table 1). Two peer promoters worked at each of the 14 study facilities.

**TABLE 1.**
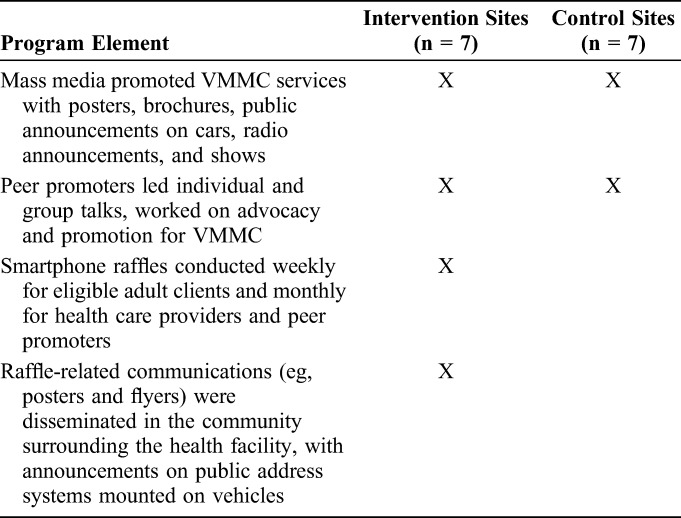
Summary of Bwana Mkubwa Intervention and Control Service Delivery Packages

**FIGURE 2.**
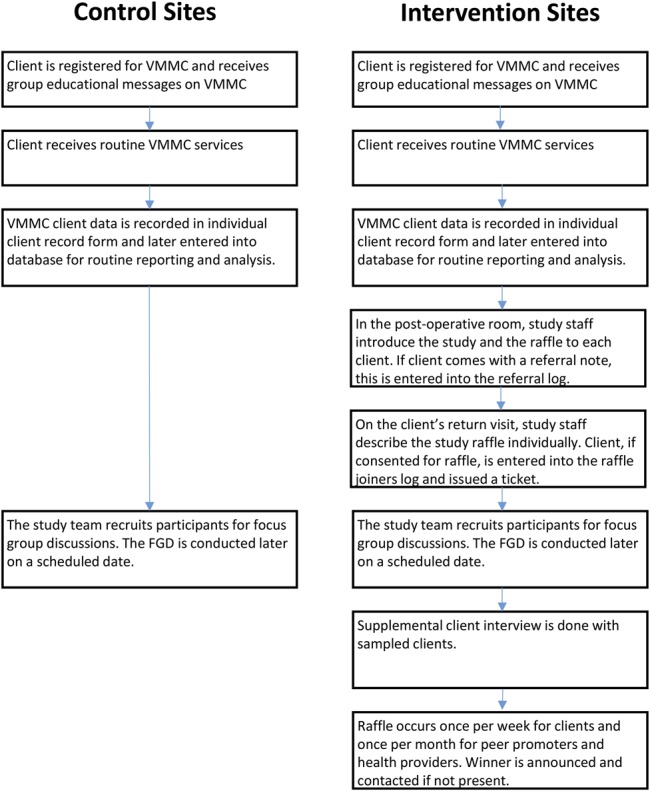
Study flow: participants at control and intervention sites.

To promote the weekly smartphone raffles, intervention facilities used public address announcements and peer promoters to inform potential clients about the raffle. VMMC clients aged 20 and above learned about the study and raffle in a group information session before the VMMC procedure and were invited to participate when they returned for follow-up (within 7 days of their procedure) (Fig. 2). The Samsung Galaxy Star S5280 phones were purchased in Tanzania for $85.60 each.

### Data Sources and Analysis

Power calculations performed in 2013 indicated that the study would be able to detect a 20% net increase in the number of circumcisions among clients aged 20 and over comparing the 2 study groups (14 facilities) during the 3-month intervention period compared with the same 3 months during the previous year. The same months were selected to account for the seasonality of VMMC care-seeking behavior.

At all sites, VMMC client medical records were entered to the routine VMMC database. Data were entered monthly into an offline system onsite and later exported to a secure, central database. Data managers checked the completeness of the forms. After reports were run, inconsistencies were examined at the facility-level and rectified. De-identified data were exported and analyzed in Stata 13 (StataCorp, 2009).

The number of VMMCs among men aged 20 and older (primary outcome) during the study period for each site and study group was compared with the period in the previous year using the generalized linear model with Poisson distribution. In a subanalysis, these data were also examined for the Iringa region, which had 3 pairs of facilities, whereas the other 2 regions had fewer facilities. We report percentage increases in VMMCs among men ages 20 and above.

Estimates were made using generalized estimating equations with an exchangeable working correlation structure and robust variance estimate. The model included an indicator variable for the periods, a study group variable, and an interaction term to create the time-by-group variables. This was a difference-in-difference analysis.

The model accounted for the clustering of data within each of the 14 facilities and also controlled for facility pair. Multivariate models also controlled for the percentages of clients aged 30 years and older who tested HIV positive at VMMC. The model's exponentiated coefficients were the estimated relative percentage increase in the average number of VMMCs in the intervention period compared with the same period in the previous year, by study group.

The study tracked raffle-related information, including the number of men who returned for follow-up, the number of men who entered the raffle, and the number of phones raffled.

### Focus Group Discussions

Study staff held 6 focus groups in Swahili with clients and 6 with peer promoters at both intervention and control sites with representation from each region (with a total of 32 peer promoters). Forty VMMC clients who returned for follow-up within 7 days of VMMC participated in focus group discussions of up to 8 participants each. The audio recordings from the focus groups were transcribed and translated into English. Transcripts were entered in Atlas-ti 7.5 software (Scientific Software Development GmbH, Berlin, Germany) for analysis. Three coders/analysts coded transcripts with a priori codes and examined for emergent themes. We grouped coded passages into themes and examined for narratives of each participant type, study group, and region. The analysts discussed findings, and wrote summary memos and placed findings into a matrix following the framework analysis approach.^27^ Final qualitative results were written to reflect all the themes identified.

The study was approved by Tanzania's National Institute for Medical Research and the Johns Hopkins School of Public Health's Institutional Review Board. Written informed consent was obtained from each participant at the intervention sites. Tanzania's Gaming Board approved the intervention of smartphone raffles. The study was registered at the Registry for International Development Impact Evaluations.

## RESULTS

### Intervention

Of 388 clients at the 7 intervention sites, 268 returned for follow-up (69%). Of these, 264 consented to be in the raffle (98.5%). Although we planned to hold 91 raffles, during some weeks, there were no raffle joiners at some sites, so only 79 raffles were held.

### VMMC Client Description

One year before Bwana Mkubwa, the mean (median) client age was 25.2 (23) in all facilities. The age increased to 27.6 (27) in the study period (*P* < 0.001) (Table 2). The percentage of VMMC clients aged 30 or older increased from 20.1% in the period in the year before the study to 30.1% during the study implementation period (*P* < 0.001). In both periods, more than a third of clients were married. The percentage of clients who tested HIV positive increased from 1.6% in the period in the year before the study to 11.9% during the study period (*P* < 0.001). In the year before the study, 84.8% of clients returned for the VMMC follow-up visit; 89.2% returned during the study period (*P* < 0.026).

**TABLE 2.**
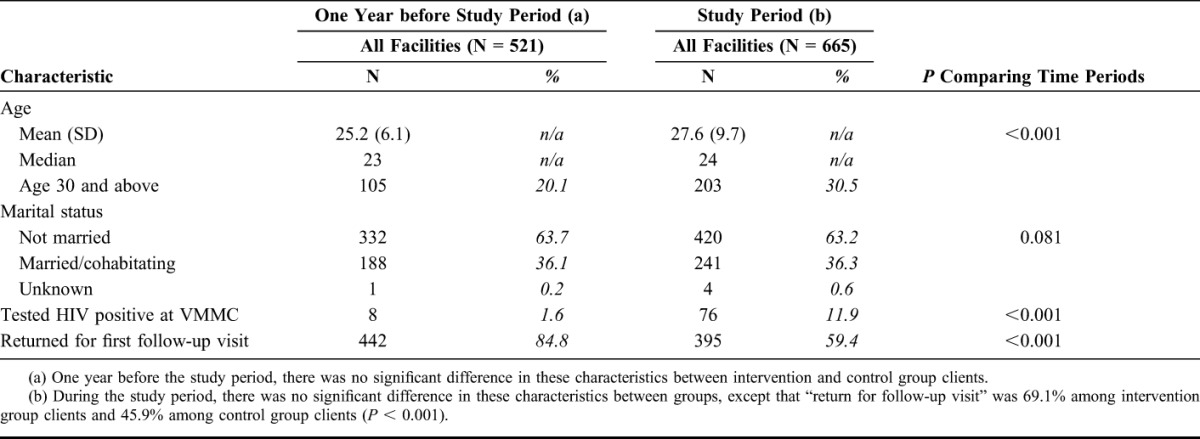
Characteristics of VMMC Clients Aged 20 Years and Above From Medical Records, One Year Before Study Period and During the Study Period

Clients returning for follow-up and entering the raffle at intervention sites differed across the 3 regions. In Iringa and Njombe, clients were more likely to be over age 30 than in Tabora (*P* < 0.001). In Iringa, clients were more likely to be engaged in farming than in the other 2 regions (*P* < 0.001). In Tabora, clients were more likely to have electricity in the house (*P* < 0.001). In all regions, more than 86% of clients reported having a mobile phone, and this was slightly higher in Tabora (96%, *P* < 0.001) (Table 3).

**TABLE 3.**
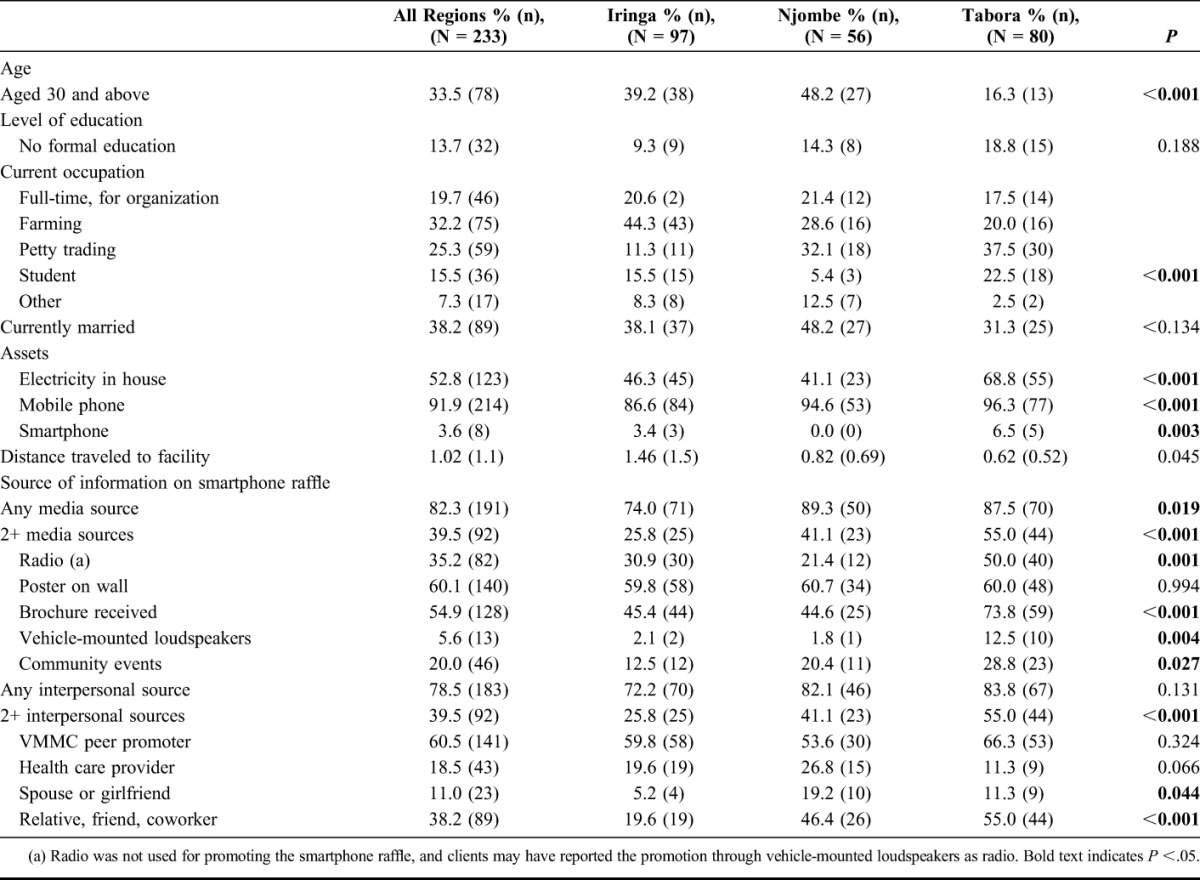
Characteristics of Surveyed VMMC Clients Who Returned for Follow-up and Entered Smartphone Raffle (Intervention Sites)

### VMMC Increases

The intervention facilities served 264 VMMC clients aged 20 and older in the same months of the prior year before the study period and 388 in the study period, an increase of 47% (Table 4). In the control group, VMMCs increased 8%, from 257 to 278. Comparisons of VMMC increases across the 2 periods and study groups were not significantly different. In the bivariate analysis and multivariable model that accounted for clustering and adjusted for facility pair, clients aged 30 years and above, and testing HIV positive, the study groups were not significantly different (*P* < 0.399).

**TABLE 4.**
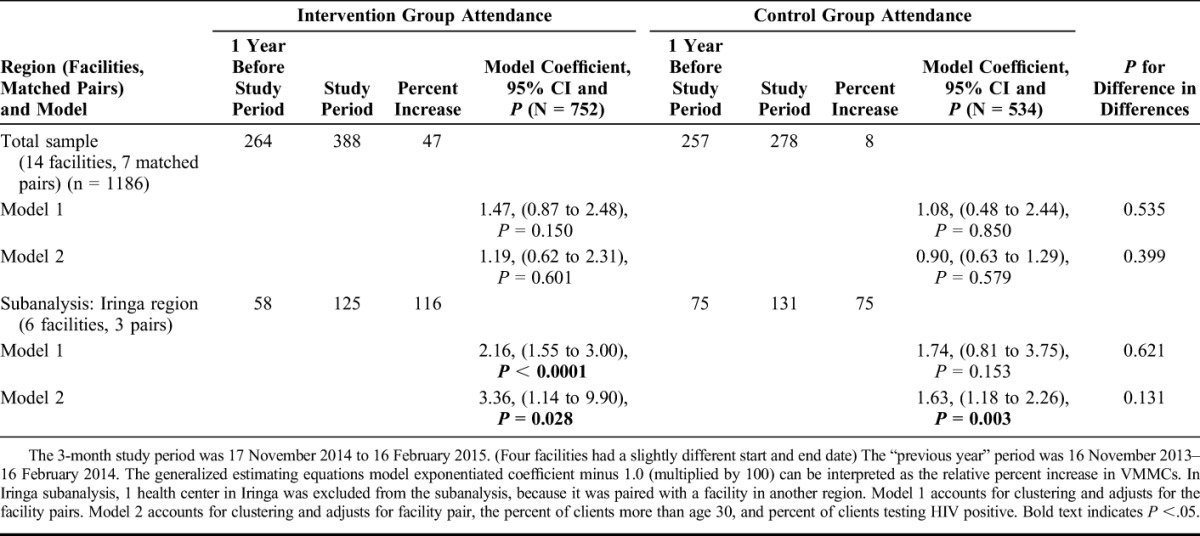
VMMC Client Attendance by Study Group at 1 Year before and During Study Period, for Iringa Region and Total Sample (N = 1186)

In the Iringa subgroup analysis, VMMCs increased 116% and 75% in the intervention and control groups, respectively. In the multivariate model, there was more than a 336% significant increase in VMMCs (exponentiated coefficient 3.36, 95% CI: 1.14 to 9.90; *P* < 0.028) in the intervention group. In Iringa, the control group had a more modest 63% significant increase in VMMCs (exponentiated coefficient 1.63, 95% CI: 1.18 to 2.26; *P* = 0.003). The two study groups' changes were not significantly different (*P* = 0.131).

### Focus Group Discussions

Peer promoters reported that the smartphone raffle succeeded in creating buzz on the street for VMMC. “*There are those who were not willing to come for circumcision earlier but now with this smartphone promotion they have shown up and they acknowledge that this phone has convinced them to show up”* (Njombe peer promoter). *“People were coming from very far for phones and by the goodness of God those from far places were almost all getting phones, so these were our good ambassadors”* (Tabora peer promoter).

Several participants in each region mentioned that the raffle raised suspicions from the community. “*[The men] have queries a lot that* ‘*you give us the service for free, medication for free and on top you give us phones?*’ *Then that's when they start thinking there should be something behind this*” (Iringa promoter). Some men wondered why the phone being given was not the older model phones that men know, such as the Nokia. Some felt the smartphone was too expensive and out of touch with the men's daily needs. One Iringa promoter explained, “*The price of smartphone is around two hundred thousand [Tanzania shillings]. With that money, you can buy 3 bicycles. The bicycle is helpful for the people living in the villages. If in the villages there is no electricity, and you give someone a smartphone, how does it help him?*” Others suggested that an incentive that all clients could receive would be preferable.

Money was most frequently recommended as an incentive for VMMC, and the amounts spontaneously suggested ranged from TSh 1000 to TSh 20,000 (approximately $0.54–$10.81). Similarly, some suggested that all VMMC clients should receive transportation reimbursement, transportation to the facility, food to take home, or farming commodities. These items would help men provide for their families while they healed from the surgery. Some clients and peer educators said they believed that a free good-quality service was incentive enough. One Tabora client at an intervention site stated, “*I do not believe that the phone is the thing which will attract people, the important thing is the service; if the service is good, people will still come.*”

## DISCUSSION AND CONCLUSIONS

The Bwana Mkubwa study had mixed results, making the relative benefit of a smartphone raffle promotion, in the context of free, high-quality services, and other behavior change communication components, challenging to interpret. Fewer than anticipated smartphone raffles were held because no raffle entrants presented during some weeks.

The study found a 47% increase in VMMC clients aged 20 and older at intervention sites during the study period compared with the same period in the previous year, and an 8% increase at the matched control sites. However, these changes were not statistically significantly different. In focus groups, some participants liked the smartphone raffle, whereas others thought that all men should receive an incentive of a modest value more related to their work or transportation reimbursement.

Comparing the 2 time periods, the number of clients aged 30 or older increased 10%, and the share of clients aged 20 and older who tested HIV positive increased from 1.6% to 11.9%. These shifts likely reflect the maturity of the VMMC program in the regions, where most of the younger men have already been circumcised and male circumcision has become an important social norm even for those who are HIV positive.

The level of scale-up of services may explain the differences in uptake of VMMC among older men in Iringa compared with the total sample. Iringa was the first region to scale up VMMC in Tanzania, with robust acceptance of VMMC services. Routine monitoring data maintained by Jhpiego and the Ministry of Health show that circumcision coverage is now at nearly 100% among 10- to 19-year-olds in Iringa and is approaching similar coverage among 20- to 24-year-olds. In contrast, the Njombe region program has struggled to overcome reluctance of promotion of VMMC among religious leaders and rumors about the government's motives for providing free circumcision. In Tabora region, the VMMC program is newer and scale-up is only approximately 50% complete. The smartphone raffle, which was implemented in the Iringa program at an opportune moment, may have played a role in helping adult men move from intention to actively seeking services, whereas the other regions may not be ready for an intervention to increase uptake among late adopters.

To our knowledge, this is the first study in which a raffle incentive was used to promote VMMC. Lotteries in other studies have had mixed results. A Lesotho study provided lottery tickets valued at either $50 or $100 to adults who tested negative for STIs; the higher payoff intervention group participants had a 21% decrease in HIV incidence over 2 years compared with the control group, which received no lottery, whereas no statistically significant decrease was found in the lower lottery group.^28^ A qualitative evaluation of a program in which a lottery was used to increase participation in workplace HIV testing found that the lottery helped to reduce stigma and engender a supportive social environment for HIV counseling and testing.^19^ Iringa region, where the raffle seemed to increase VMMCs significantly, might have a more favorable social environment for VMMC seeking, greater desirability of smartphones, and/or a more favorable perception of the value of the smartphone in offsetting lost wages.

### Study Limitations

A limitation of this study is that the rigorous evaluation of the smartphone raffle intervention was not preceded by formative research to determine acceptability of the planned intervention among the local population. An implementation period longer than 3 months would have yielded higher VMMC uptake and a higher sample size with possibly higher power to detect statistical differences.

At the project design stage, after looking at monthly VMMC volumes with data from February to June 2013, it was determined that 14 facilities would provide sufficient power given the study objectives and with the parameters and assumptions used. VMMC volumes from February to June 2013 were higher than those found during the study period (November 2014–February 2014), possibly for 2 reasons. The period includes the farming season during which men are less able to miss work for the post-VMMC healing period and end-of-year holidays, in which men may not wish to be abstinent during the post-VMMC healing period. Another reason for lower VMMC volumes in 2014–2015 may have been saturation, as many men in the community would have already been circumcised by 2014 and those attracted to VMMC would be late adopters.

Qualitative data were not collected from men in the community who did not seek VMMC or did not return for follow-up. However, the focus group discussions with peer promoters described interactions with men who refused VMMC services.

This study contributes to the growing evidence on how to promote and incentivize VMMC service uptake among adult males in regions of sub-Saharan African countries with high HIV burden. A smartphone raffle might increase VMMC uptake in some settings by helping late adopters move from intention to action; however, there is no recommendation for this intervention more generally.
